# Current Mechanistic Understandings of Lymphedema and Lipedema: Tales of Fluid, Fat, and Fibrosis

**DOI:** 10.3390/ijms23126621

**Published:** 2022-06-14

**Authors:** Bailey H. Duhon, Thien T. Phan, Shannon L. Taylor, Rachelle L. Crescenzi, Joseph M. Rutkowski

**Affiliations:** 1 Department of Medical Physiology, Texas A & M University College of Medicine, Bryan, TX 77807, USA; bd009444@tamu.edu (B.H.D.); ttphan@tamu.edu (T.T.P.); 2 Department of Biomedical Engineering, Vanderbilt University School of Engineering, Nashville, TN 37232, USA; shannon.l.taylor@vanderbilt.edu; 3 Department of Radiology and Radiological Sciences, Vanderbilt University Institute of Imaging Sciences, Vanderbilt University Medical Center, Nashville, TN 37232, USA

**Keywords:** lipedema, lymphedema, vascular disease, obesity, adipogenesis, inflammation, pain, fibrosis, extracellular matrix

## Abstract

Lymphedema and lipedema are complex diseases. While the external presentation of swollen legs in lower-extremity lymphedema and lipedema appear similar, current mechanistic understandings of these diseases indicate unique aspects of their underlying pathophysiology. They share certain clinical features, such as fluid (edema), fat (adipose expansion), and fibrosis (extracellular matrix remodeling). Yet, these diverge on their time course and known molecular regulators of pathophysiology and genetics. This divergence likely indicates a unique route leading to interstitial fluid accumulation and subsequent inflammation in lymphedema versus lipedema. Identifying disease mechanisms that are causal and which are merely indicative of the condition is far more explored in lymphedema than in lipedema. In primary lymphedema, discoveries of genetic mutations link molecular markers to mechanisms of lymphatic disease. Much work remains in this area towards better risk assessment of secondary lymphedema and the hopeful discovery of validated genetic diagnostics for lipedema. The purpose of this review is to expose the distinct and shared (i) clinical criteria and symptomatology, (ii) molecular regulators and pathophysiology, and (iii) genetic markers of lymphedema and lipedema to help inform future research in this field.

## 1. Introduction

Lymphedema and lipedema are often misunderstood diseases that remain understudied and underappreciated. Lymphedema results from a genetic or acquired lymphatic deficiency causing tissue fluid accumulation, inflammation, and adipose tissue expansion [1]. The pathological adipose expansion in lipedema, however, arises from an etiology that is not yet well defined but also exhibits tissue fluid accumulation and aspects of connective tissue remodeling [2]. This combination of altered homeostasis of fluid and fat in lymphedema and lipedema adds to the difficulty in distinguishing these diseases. It is critical to study their molecular and genetic regulators to inform their differential diagnoses and improve the long-term outcomes of physical and emotional distress in patients with lymphedema or lipedema who suffer from a dearth of treatment options when left undiagnosed and mismanaged.

Herein, we explore the current mechanistic understandings of lymphedema and lipedema to better expose their distinct and shared (i) clinical criteria and symptomatology, (ii) molecular regulators and pathophysiology, and (iii) genetic causes or indicators of disease. In each section, we outline the salient features known about lymphedema and compare these to several identified or rational targets in lipedema. To help elucidate promising avenues of research into these understudied diseases, this review focuses on a trifecta of disease manifestations in lymphedema and lipedema: fluid, fat, and fibrosis.

## 2. Clinical Criteria and Symptomatology

### 2.1. Lymphedema

Lymphedema is a debilitating chronic disease estimated to affect 140–250 million people worldwide and approximately 1 out of every 1000 people in the United States alone [3]. It can be classified by etiology, distinguishing primary cases (hereditary, congenital, and idiopathic) from secondary lymphedema. Symptom onset of primary lymphedema can be early in life (congenital), around puberty (praecox), or later in life (tarda) [4]. Secondary lymphedema can arise post-operatively from cancer therapies involving lymph node dissection or radiation. The most common form of cancer-related lymphedema is due to breast cancer therapies, while lower-extremity secondary lymphedema also has high incidence rates due to gynecological cancer (20–40%), sarcoma (30%), melanoma (16%), and genitourinary cancer (10%) [5,6]. More research is needed regarding lymphedema risk, onset, and development of this heterogeneous disease [7,8].

Lymphedema clinical staging applies a standard set of criteria to grade the severity of disease (Table 1). Lymphedema-staging criteria considers a patient’s clinical history, limb volume, edema status, as well as skin texture [9]. Lymphedema stage 1 exhibits edema that can resolve with rest, while later stages may exhibit a unilateral or bilateral persistent swelling. Lymphatic deficiency of the distal extremities is clinically noted by an inability to pinch the skin on the dorsum of the foot, which is referred to as a positive Kaposi–Stemmer sign [10]. Patients may also present with skin changes, such as dimpling, commonly referred to as peau d’orange, along with pitting edema. In advanced stages of lymphedema, the skin texture changes, often without pitting, indicative of skin thickening and tissue fibrosis [11]. Moreover, lymphedema skin thickening can advance to a hyperkeratotic state with cobblestone-like papules or lichenification [12]. Tissue fibrosis in lymphedema is almost always accompanied by subcutaneous adipose tissue (SAT) expansion [13,14,15,16].

While lymphedema-staging criteria does not consider pain or limb function, patients may report diffuse pain, limb heaviness, and numbness that impact the patient’s quality of life and lower-extremity function [3,9,19,20,21,22]. Monitoring pain and function of patients with lymphedema deserves further attention, especially as this may demonstrate efficacy of therapeutics, such as complete decongestive therapy and lympho-venous anastomosis [19,20,23].

### 2.2. Lipedema

Lipedema is a distinct disease recognized by The American College of Cardiology with unique diagnostic and treatment considerations separate from that of lymphedema and other diseases with extremity swelling [24]. However, the etiology of extremity swelling in lipedema remains unknown and the disease continues to be misdiagnosed. Lipedema incidence is in fact estimated from the rate of misdiagnosed patients treated for lymphedema and could be as high as 6–11% of women in the population [25,26]. Likewise, lipedema is typically misdiagnosed as obesity and only differentiated after attempts at weight loss have minimal effect on lower-extremity girth in patients afflicted with lipedema [2,18,27].

The diagnosis of lipedema requires training and the knowledge of lipedema to identify disease features during a physical clinical exam as well as extensive knowledge of the patient’s medical history [2]. Clinical staging of lipedema is based on the morphological presentation of the legs, especially the skin and adipose tissue deformations (Table 1). Recently updated lipedema diagnostic criteria consist of gynoid SAT accumulation that is specifically resistant to weight-loss therapy, bilateral lower-extremity swelling that is persistent despite elevation, and palpable subcutaneous nodules [2]. These criteria aim to differentiate lipedema from non-lipedema obesity, where adipose tissue distribution is usually android and responsive to conventional weight loss methods and does not present with edema or pain. Edema can be present in areas affected by lipedema and is non-pitting where fibrotic tissue turgor impedes the pitting test [2,28]. Unlike lymphedema, lipedema swelling typically spares the hands and feet, and therefore, the Kaposi–Stemmer sign is not pathognomonic [10]. Lipedema is additionally classified by “type” based on the location of disproportionate lower-extremity SAT deposition. Type indicates that SAT extends through the I. hips, II. knees, or III. ankles, while type IV. indicates arm involvement, and type V. indicates only calf involvement [29]. Similar to lymphedema staging, pain is not considered in current lipedema-staging guidelines although it correlates with lipedema disease severity [30].

While clinical features of fluid, fat, and fibrotic tissue deposition are shared with lymphedema, other clinical components of lipedema are distinct. The most common symptoms of lipedema following SAT expansion are easy bruising and significant pain in the affected areas [2,18,30,31,32,33,34]. These features are distinct from lymphedema: blood capillary fragility is not an aspect of the primary etiology in lymphedema, and lipedema-associated pain is more chronic and severe than reported lymphedema pain. Pathogenic pain, akin to neuropathy, may be present in up to 90% of lipedema patients independent of their BMI; this has the potential to further distinguish lipedema from obesity if objectively characterized [35]. Interestingly, a large number of patients with lipedema (approximately 50%) display joint hypermobility, which can be clinically assessed via the Beighton criteria or musculoskeletal questionnaires. However, this feature is not well-studied and deserves further investigation. [2,30,36,37,38]. Finally, lipedema almost exclusively occurs in females, has a strong familial history, and symptoms onset around periods of hormonal change (i.e., puberty, pregnancy, menopause), suggestive of estrogen-driven disease mechanisms and heritable traits [39,40].

### 2.3. Summary

**Figure 1:**
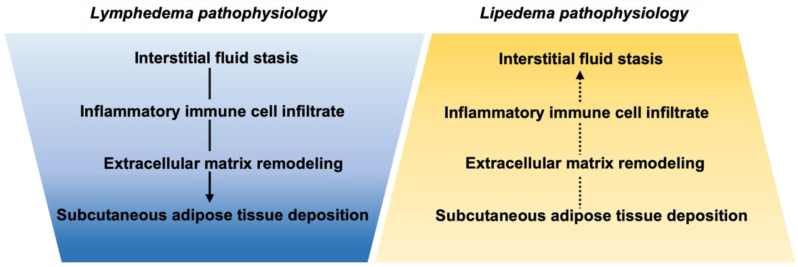
Pathophysiological features of lymphedema and lipedema are represented graphically to emphasize (i) the overlapping pathologies involved in each disease, and yet (ii) their different prominence in disease progression. Pathologies which are most prominent are graphed in the widest section, with fluid being most prominent in lymphedema and adiposity most prominent in lipedema. The time course of these pathologies is potentially reversed, represented by a solid arrow for the well-established disease progression of lymphedema, and a dashed arrow in reverse order for lipedema where adipose tissue or extracellular matrix dysfunction could give way to inflammation and an increased immune response.

Lipedema and lymphedema have distinct staging criteria that are not exclusive of each other (Table 1). Lipedema and lymphedema share certain clinical features, including fluid (edema), fat (adipose expansion), and fibrosis (extracellular matrix remodeling); yet, they diverge on the time course of their development (Figure 1). For instance, both lipedema and lymphedema present with pathologic SAT expansion that develops into fibrosis. Fibrosis indicates advanced stages of lymphedema (stage 2), while adiposity with fibrotic tissue texture is prominent early in lipedema (stage 1). Likewise, tissue fluid is the prominent feature of lymphedema onset and in lipedema may become more prominent in advanced stages [30]. Distinct clinical symptoms of lipedema include easy bruising, pain, and joint hypermobility that implicate more primary microvascular, neuronal, and connective tissue involvement in mechanisms of lipedema. Next, we discuss and compare the potential molecular regulators and pathophysiology of lymphedema and lipedema relevant to their shared clinical features.

## 3. Molecular Regulators and Pathophysiology

### 3.1. Lymphedema

Although lymphedema can arise from either primary or secondary etiologies, the pathogenesis is similar resulting from ineffective or absent lymphatic fluid transport [41]. Prior to overt limb swelling, interstitial fluid accumulation occurs as a direct result of local or downstream lymphatic insufficiency. This insufficiency also results in the accumulation of macromolecular proteins normally transported by lymph including hyaluronan, lipoproteins, tissue antigens, and cellular metabolites within the extracellular space throughout the region of lymphatic obstruction and afferent tissues [42]. The subsequent lymphedema pathophysiology involves a sustained inflammatory response that drives fluid deposition towards tissue fibrosis and adipose expansion [43,44]. These prominent pathophysiological mechanisms (Figure 1) are outlined in the remaining sections. 

Lymphedema occurs when the capacity of lymphatic vessels to transport interstitial fluid fails to meet the demand of physiologically normal levels of blood capillary extravasation. This may be due to, in the case of primary lymphedema, aberrant lymphatic anatomy or function that may include lack of lymphatic capillaries or valve formation, lymphatic hyperplasia, malformations, leaky vessels, or impaired pumping (among others) [45]. In secondary lymphedema, typically the large collecting lymphatic vessels or nodes have been damaged, compressed, or severed due to infection, obesity, or surgery, respectively. In either case, the potential result is tissue lymph stasis which alters interstitial pressures, protein concentrations, and local cytokine levels [46,47]. Lymphatic vessels not only transport fluid, but also immune cells, and thus, prolonged lymph stasis induces an inflammatory response [48]. Not all patients with lymphatic disruption demonstrate lymphedema, however, such that the biochemical and biomechanical perturbations of infection or inflammation in the tissue may serve as a periodic driver of clinical lymphedema [49]. 

The inflammatory response in lymphedema typically entails increased expression of a host of cytokines and chemokines and increased immune cell accumulation and activation. There are various immune cell populations that may play a role in the inflammatory response driving lymphedema. For example, in a mouse model of lymphedema, the tissue demonstrated increased CD4+ T cell numbers and T-helper 2 (Th2) differentiation [50]. These cells play a role in the lymphedema pathogenesis, as inhibiting Th2 differentiation decreases fibrosis and improves lymphatic function [50,51]. Human tissue samples from limbs with lymphedema confirm elevated CD4+ T cell quantities that correlate with lymphedema stage, and thus, restoring normal T cell function may improve lymphedema [52]. Topically applied tacrolimus, an immunosuppressant that reduces T cell numbers, successfully ameliorated both lymphedema swelling and tissue fibrosis in mice and may be translatable to humans [53]. Animal models of lymphedema have also demonstrated that inflammation-induced CD4+ T cells stimulate vascular endothelial growth factor C (VEGF-C) expression [54]. This potent lymphangiogenic factor drives lymphatic hyperplasia in the tissue [54]. Increased CD4+ T cells also correlate with lymphatic vessel remodeling and collector vessel impairment in disease progression [55]. Collectively, these findings illustrate an important and necessary role of CD4+ T cells in the inflammatory response in lymphedema.

Beyond immune cell activation, there are dramatically increased levels of pro-inflammatory cytokines in lymphedema tissue [56]. These include, but are not limited to IL-2, IL-4, IL-6, IL-8, IL-13, MCP-1, and TNFα [46,49,56]. Additional inflammatory mediators, including certain fatty acids and leukotriene B_4_, have also been detected in excess within adipose tissue of lymphedematous limbs [57,58]. Moreover, this inflammatory response can also be measured in circulation, with increased circulating cytokines TNFα, IL-6, IL-8, and MCP-1 measured in lymphedema patients [59,60]. Elevated platelet factor 4 (PF4), an inflammatory cytokine that also promotes blood coagulation, was found in blood-borne exosomes of patients with lymphedema and other lymphatic conditions [61]. Gene expression of inflammatory markers can be reduced in lymphedema patients that undergo decongestive therapy, and these pro-inflammatory cytokines can also act as therapeutic targets [62]. For example, neutralizing antibodies targeting IL-4 and IL-13 successfully decreased Th2 cytokines and reduced epidermal thickness [49]. Additionally, in both animal models and patients with lymphedema, inhibiting leukotriene B_4_ reduced skin thickness, demonstrating the significance of inflammation in lymphedema symptoms and its potential as a therapeutic target [63,64].

Sustained inflammatory response associated with lymphedema leads to tissue remodeling towards fibrosis, characterized by an increase in collagen deposition accompanied with adipose and connective tissue overgrowth [48,65]. Immune cell recruitment (macrophages, fibroblasts, CD4+ T cells, and Th2 cells) has also been shown to facilitate adipose deposition and fibrosis in lymphedema [50,51,66,67,68]. Specific populations of immune cells have enhanced roles, with CD4+ T cell activity also being implicated in extracellular matrix (ECM) remodeling. In addition, Th2 cells promote fibroblasts and macrophages, which leads to increased collagen, cytokine, and matrix metalloproteinase (MMP) production [52,65,66,69]. Increased levels of MMP-9, for example, were measured in conjunction with fibrosis in lymphedema patients treated for head and neck cancer [70]. Transforming growth factor-B1 (TGF-β1), a potent pro-fibrotic cytokine, is elevated in lymphedematous tissue in both mice and patients [71,72,73]. Multiple studies have demonstrated that neutralization of TGF-β1 can diminished fibrosis and ameliorate lymphatic function [73,74]. 

While adipose expansion clearly occurs, the mechanisms driving it and theadipose pathophysiology in lymphedema are not as well-characterized as the fluid component of the disease. Imaging of body composition in patients with lymphedema identified SAT deposition in the affected limbs with some intramuscular fat infiltration [14,75,76,77,78]. SAT expansion may be indicative of inflammation and fibrosis rather than metabolic imbalance, though obesity exacerbates lymphedema in experimental models [79,80]. Additionally, vascular function in relation to SAT deposition, in particular potential clearance by lymphatics, deserves further attention. Lymphatic dysfunction in genetic mouse models, for instance, can drive subcutaneous adipose expansion [81,82]. Adipose expansion can also impede lymphatic function, creating a potentially vicious cycle in lymphedema [79,80,83]. These provocative mechanistic possibilities would be interesting to observe in human disease with further development of noninvasive lymphatic-imaging technologies [84]. This intimate connection between lymphatic function and adipose tissue is thought be part of the pathogenesis of lipedema. 

### 3.2. Lipedema

The etiology of lipedema remains unclear and has incited a recent *Call to Action* [35]. Various hypotheses have been proposed regarding the pathogenesis of the primary symptom of lipedema: lower-extremity SAT expansion. It is important to note that SAT expansion in lipedema behaves differently than in obesity, with characteristic resistance to metabolic interventions, implying an alternative etiology [85,86]. Rational hypotheses incorporate adipocyte dysfunction and adipogenesis, tissue inflammation, altered ECM components, or microvascular dysfunction leading to fluid accumulation in lipedema. These hypotheses do not leave out the possibility that pathogenesis is multifactorial. Here, we will discuss recent findings in the pathophysiology of lipedema in the order of prominence in the disease: fat, fibrosis, and fluid accumulation (Figure 1).

SAT expansion in lipedema occurs with adipocyte hypertrophy [87,88]. Additionally, several studies have provided evidence of adipogenesis in lipedema via proliferation of adipose-derived stem cells (ASCs). CD34+ and Ki67+ ASCs were found to be colocalized with areas of macrophage residency and adipocyte necrosis [89]. ASCs derived from lipedema tissues exhibited a distinct gene expression compared to controls [88]. Similar but slightly elevated adipogenic gene expression between lipedema and control samples was noted in an in vitro 3D matrix of ASCs (so-called spheroids), characterizing the adipogenic potential in lipedema pathology [90]. In lymphedema, adipose deposition occurs with persistent inflammation and thus, postulates whether a source of inflammation is also present in lipedema. Similar to lymphedema, inflammatory markers in lipedema include immune cell infiltrates in the SAT. Specifically, elevated macrophages surround adipocytes in lipedema SAT forming adipose crown-like structures were noted [87]. CD45+ lymphocytes and CD68+ macrophages were also observed in lipedema adipose tissue [88,89] and skin [91]. However, this infiltrate does not appear to contain the same T cell involvement identified as a key feature seen in lymphedema [88,89,91]. 

While immune cell recruitment regulates fibrosis formation in lymphedema [50,51,66,67,68], this mechanism is less clear in lipedema. A fibrotic pathology of the ECM in lipedema tissue has been identified in two independent cohorts [88,89]. Increased fibrosis and changing composition of the ECM is characteristic of and can further drive adipose tissue dysfunction [92,93,94]. Fibrotic ECM could also be a source of elevated tissue sodium content in lipedema leg SAT observed on molecular-imaging studies [95,96]. Comparative studies of imaging to histology should be carried out in lipedema and lymphedema to improve our understanding of SAT composition and function in the setting of fibrosis. An intriguing hypothesis for lipedema pathogenesis involves improper ECM remodeling, whereby uncoupling of the MMP-14-caveolin 1 (CAV1) axis in adipocytes may cause aberrant matrix processing, resulting in hypertrophic SAT expansion [97]. Initial studies into this pathophysiologic mechanism found slightly lower expressions of MMP-2, -9, and -11 in a cohort of patients with lipedema compared to controls, with no significant difference in ECM fibronectin or collagen [90]. Changes to the ECM or the compliance of connective tissue would be interesting to study in relation to patient symptomatology of joint hypermobility, as well as findings of reduced elasticity of the skin [98] and aorta [99] in some patients with lipedema. Dietary supplements, such as nattokinase, selenium, and diosmin, with suggested anti-inflammatory and anti-fibrotic properties have demonstrated some efficacy in lipedema patients but require further study [31]. The degree of ECM changes, whether they are pervasive in all lipedema patients or a subset, and whether the ECM is a primary etiology of lipedema pathogenesis remains unclear and deserves further study.

Another potential inflammatory route in lipedema is via interstitial fluid accumulation resulting from microvascular dysfunction. Interstitial fluid and tissue volume homeostasis are disrupted in lipedema, with evidence of interstitial fluid accumulation [91] and tissue sodium and adipose deposition [95,96]. Indeed, lymphatic bulk transport is compromised in lipedema [100,101,102,103,104], lending clinically significant evidence of lymphatic disease. Recent evidence of elevated platelet factor 4 (PF4) in patients with lipedema, a circulatory marker shared in patients with lymphedema, further supports the lymphatic dysfunction-adipose expansion hypothesis [61]. VEGF-C levels are increased in lipedema, but corresponding changes to the lymphatic architecture are largely absent [87,105]. Unlike in lymphedema, there is stronger evidence for a blood capillary component to lipedema. Research has demonstrated microangiopathy in lipedema, including patient reports of frequent hematoma [30], imaging of vascular permeability [106], and capillary fragility [107]. Endothelial glycocalyx porosity, if disrupted in lipedema, could induce excess fluid extravasation and inflammation [91,95,108]. A histological study revealed an increased number of dilated blood capillaries in lipedema SAT [87]. Thus, current evidence supports blood and/or lymphatic microvascular contributions to interstitial fluid in lipedema.

### 3.3. Summary

In summary, lipedema pathophysiology is significantly less understood than that of lymphedema. Like in lymphedema in which interstitial fluid accumulation can induce inflammation and fibrosis [109], interstitial fluid accumulation could drive inflammation and subsequent tissue remodeling responses in lipedema. However, unlike lymphedema where the lymphatic etiology is clearly identified and immune cell involvement is increasingly well-characterized, the origins of fluid collection in lipedema are less clear. The source of interstitial fluid in lipedema could be lymphatic or blood capillary dysfunction, and likely involves a more prominent role of adipocyte biology, extracellular matrix composition, and hormonal activity. Therefore, while there are many similarities between lipedema and lymphedema, key differences between the two diseases may help to identify the distinct pathogenesis of lipedema. Genetic studies may hold the key to identifying their molecular underpinnings.

## 4. Genetic Causes or Indicators

### 4.1. Lymphedema

The development of primary lymphedema is likely due to altered genetics, but only around one-third of all cases have a known mutation [110]. Known mutations that lead to primary lymphedema, often identified and characterized through familial segregation analyses, can be autosomal dominant or autosomal recessive. These mutations are most often in genes associated with embryological vascular and lymphatic development, post-fetal lymphangiogenesis, lymphangiogenic-associated pathways, or extracellular matrix composition. All result in some degree of lymphatic insufficiency and lymphedema in both human and genetic murine models. These mutations have been elegantly compiled and reviewed elsewhere, with a few noteworthy selections provided here: [1,110,111]. 

The lymphatic vasculature begins to develop from the endothelial cells in the cardinal vein that upregulate the transcription factor Prox-1 [112,113]. Prox-1 then regulates expression of *SOX18*, *FOXC2*, and *VEGFR3* [112,113]. Not surprisingly, mutations in these genes, as well as other genes critically necessary for blood vasculogenesis, were the first to be identified in familial lymphedema. Several genes specifically have been associated with named lymphatic diseases, such as *SOX18* in hypotrichosis-lymphedema-telangiectasia [114], FOXC2 in lymphedema-distichiasis [115], and *VEGFR3* in Nonne–Milroy disease [116,117]. Other genes have been implicated in a variety of lymphangiogenic processes and include adhesion proteins (*PIEZO1* [118], *SVEP1* [119], and CELSR1 [120]), growth factors (*ANGPT2*-*TIE2* [121] and *HGF* [122]), transcription factors (*GATA2* [123]), receptor proteins (*EFNB2* [124]), and Notch-signaling genes [125]. 

It has been suggested that patients who develop secondary lymphedema following surgery and/or lymphadenectomy may have a genetic predisposition that places them at-risk for lymphedema, notably mutations in *HGF* and *VEGFR3* [8,126,127]. This predisposition was elegantly visualized when it was identified that the contralateral arm of breast cancer lymphedema patients also demonstrates lymphatic impairment [128]. Importantly, lymphedema is two to three times more likely to be observed in females compared to males, suggesting a sex-linked genetic component to lymphatic mechanisms, such as VEGFR-3 signaling [129,130,131,132]. More research into the genetic profile of patients who develop lymphedema would help provide appropriate risk-assessment for those undergoing cancer therapies.

### 4.2. Lipedema

Appreciation for lipedema as a unique disease has increased efforts to identify specific causative genes. Any genes identified may also help researchers to better understand the disease’s underlying pathogenesis. The prevalence of lipedema in familial history suggests heritability and several studies have examined pedigrees in search of the method of heritability and inheritable phenotypes [40]. It is thought to pass from parent to offspring in either X-linked dominant inheritance or autosomal dominant inheritance with sex limitation, with the latter being more likely [40]. A mutation in one such gene, the *AKR1C1* gene that encodes for aldo-keto reductase, has been identified in a family with clinical criteria for lipedema, including adipogenesis and SAT deposition (and, interestingly, elevated progesterone) [133]. Another familial study found a mutation to the *PIT1* gene for growth- and sex-hormone expression in those with lipedema-like symptoms and not in phenotypically normal individuals [134]. 

Candidate genes may arise from animal models and human diseases that resemble features of lipedema. In murine models, *VEGFR3* mutations associated with development of lymphedema in humans have immature lymphatic vessels, unaffected blood vessels, and thickened SAT layers [135]. These models and others with lymphedema-causing mutations may provide insight into the coupling of SAT expansion and lymphatic microvascular dysfunction. In humans, William’s syndrome is a genetic disease with a lipedema-like phenotype of lower-extremity SAT deposition in approximately 25% of cases [136]. The genetic origins of William’s syndrome involve the *ELN* gene that encodes for elastin [137]. *ELN* gene mutations are thought to be responsible for the vascular and connective tissue impairments in William’s syndrome that are also associated with excess adipose deposition [136,137,138]. The anecdotally-reported high incidence of hypermobility in lipedema also hints at a possible connection to non-vascular Ehlers–Danlos syndrome which is driven by mutations in several collagen or ECM-related genes [139].

Gene-sequencing studies are underway in multiple centers specializing in lymphedema to identify potential variations in lipedema. Notably, next-generation sequencing has elucidated 21 deletion variants in 12 genes shared by a subset of patients with lipedema; these genes were associated with adipogenesis, adipocyte biology, and metabolism consistent with aberrant adipose tissue remodeling and function and with the heterogenous nature of lipedema [140]. Similarly, a recent genome-wide association study of 130 lipedema patients identified several single nucleotide polymorphisms, and the authors highlighted those associated with lipoma formation and hormone synthesis as mechanistically relevant [141]. Further studies in well-characterized cohorts with lipedema should provide more insight into the genetic basis of lipedema. 

### 4.3. Summary

Genetic effectors of lymphedema and lipedema may converge in future studies; however, mutations known to cause primary lymphedema have not yet been identified in lipedema although much work remains in this area. In fact, genetics have only been used in lipedema to date to rule out other conditions [142]. With identification, further studies manipulating these genes in animal models may recapitulate lipedema features to explore potential modes of pathogenesis and therapeutics. 

## 5. Summary and Call for Further Research

Our current mechanistic understanding of lower-extremity lymphedema and lipedema is only beginning to shed light on the complex physiology involved in these diseases. Their clinical features overlap in similar presentation of limb swelling from fluid, fat, and fibrosis, though also differing with distinct features of pain and easy bruising often present in lipedema. The diagnosis and understanding of lymphedema have become increasingly granular through research and clinical reports, and we anticipate a similar trajectory in the field of lipedema. Many questions remain, however, as to the mechanisms of lipedema. To what extent is it a lymphatic disease? Is it a connective tissue disease? What drives lipedema-associated pain? Which genetic pathways give rise to which phenotypes of lipedema? A unique opportunity exists by studying lymphedema and lipedema to better understand the interplay between tissue fluid, fat, and fibrosis in disease. Research regarding the molecular regulators of pathophysiology and potential genetic markers may help to elucidate the complex etiology of lipedema, identify additional objective diagnostic criteria to distinguish it from lymphedema, and provide targetable and measurable therapies to improve the lives of patients suffering physically and emotionally from these diseases. 

## Figures and Tables

**Table 1:** Clinical criteria for lymphedema and lipedema staging by presentation.

	Lymphedema ^1^	Lipedema ^2^
**STAGE 0**	No observable limb swelling or pitting; limb is at risk for lymphedema following lymph node removal	N/A
**STAGE 1**	Reversible lymphedema with symptoms of pitting edema; elevation reduces edema	Limbs exhibit smooth skin with enlarged subcutaneous adipose tissue
**STAGE 2**	Stage 1 criteria plus fibrosis; pitting may be present; elevation does not fully reduce edema	Uneven skin with indentations in the adipose tissue and larger mounds of tissue able to be seen or felt
**STAGE 3**	Tissue is hard and pitting can be absent; skin changes such as thickening and hyperpigmentation	Large extrusion of adipose tissue causing deformations especially around the thighs and knees

^1^. Executive Committee of the International Society of Lymphology, The diagnosis and treatment of peripheral lymphedema: 2020 Consensus Document of the International Society of Lymphology. *Lymphology* 2020 [17]. ^2^. Herbst, K.L. Rare adipose disorders (RADs) masquerading as obesity. *Acta. Pharmacol. Sin.* 2012 [18].

## Data Availability

Not applicable.
